# Image Analyzer-Based Assessment of Tumor-Infiltrating T Cell Subsets and Their Prognostic Values in Colorectal Carcinomas

**DOI:** 10.1371/journal.pone.0122183

**Published:** 2015-04-15

**Authors:** Younghoon Kim, Jeong Mo Bae, Gang Li, Nam Yun Cho, Gyeong Hoon Kang

**Affiliations:** 1 Department of Pathology, Seoul National University College of Medicine, Jongno-gu, Seoul, Korea; 2 Laboratory of Epigenetics, Cancer Research Institute, Seoul National University College of Medicine, Jongno-gu, Seoul, Korea; Memorial Sloan-Kettering Cancer Center, UNITED STATES

## Abstract

To find useful tools to evaluate the prognosis in colorectal carcinoma (CRC) patients, we investigated the prognostic values of tumor-infiltrating T lymphocyte subsets according to intratumoral subsites as well as clinical or molecular characteristics. Immunohistochemistry for CD8, CD45RO, and FOXP3 was performed, and densities of the T cell subsets in each tissue microarray core (cells/mm^2^) were measured by image analysis. In the training set (n = 218) of CRC, T cell subset densities in the invasion front were more strongly associated with patient outcome than those in the tumor center. In the validation set (n = 549), T cell subset densities in the invasion front were evaluated. Univariate analysis showed that all three T cell subset densities were significantly associated with longer progression free survival and overall survival time (*p* < 0.001). In multivariate analysis, a high CD45RO density correlated independently with longer progression free survival (*p* = 0.011) and overall survival time (*p* = 0.007) in CRC patients, regardless of tumor location or adjuvant chemotherapy status. Our results showed that CD45RO density in the invasion front was the only independent prognostic factor regarding CRC. However, CD8 and FOXP3 densities were also independent prognostic factors in certain clinical settings. Thus, image analysis of tissue microarray cores in the invasion front of CRC could be used as a valid method for evaluating the prognostic significance of T cell subset densities.

## Introduction

Colorectal cancer (CRC) is the second most common malignant tumor worldwide and is one of the most prevalent cancers in Western society [1,2]. Despite the high mortality of CRC [1], advance in accurate prediction of patient prognosis is discouraging. Therefore, identifying potential prognostic factors is essential in understanding tumor progression of CRC and identifying novel therapeutic targets of the disease [3].

Cancer cells are known to express tumor-associated antigens (TAAs) and chemokines, which become targets of the T cell-mediated immune response [4,5]. Tumor-infiltrating T lymphocytes (TILs) mediate adaptive immunity and are associated with prognosis in solid tumors, such as in liver, breast, lung, stomach, and colon [6–8]. T cells can be subdivided into functional subtypes, including cytotoxic CD8+ T cells (CTLs), memory CD45RO+ T cells [9–11], and regulatory T cells (Tregs; forkhead box P3 [FOXP3]) [12].

Regarding CRC, CD8+ T cells are essential in recognizing and lysing the malignant cells in microenvironment [13]. High infiltration of CD45RO+ T cells has been associated with increased expressions of T-helper1 (Th1) and cytotoxicity-related genes in early-stage CRC [14]. FOXP3+ T cells inhibit Th1 and T-helper2 (Th2) as well as T-helper17 (Th17) in CRC [15]. However, questions still remain as to which T cell subset in CRC bears the prognostic information [16].

Accumulating evidence suggests that cancer cells in different subsites within tumors, including the tumor center and the invasion front, express different molecular and pathological characteristics [16,17]. In CRC, tumor cells in the invasion front show more aggressive behavior than those in the tumor center [17,18]. It could thus be hypothesized that neoplastic cells might express different TAA and chemokine patterns according to intratumoral subsites. However, most previous studies did not distinguish the tumor center from the invasion front when evaluating the correlation between T cell subsets and patient survival in CRC [14,16,19–26]

The aforementioned findings prompted us to investigate which T cell subset is a potential marker of prognosis in CRC and which intratumoral subsite should be used for such a purpose. Therefore, the present study extended previous studies using immunohistochemical tissue microarray analysis and image analysis applied to a large number of CRC cases (n = 767) to elucidate the prognostic significance according to type of individual T cell subset (CD8+, CD45RO+, and FOXP3+ phenotypes) densities and intratumoral subsite (tumor center or invasion front). In addition, we examined clinical and molecular variables, including tumor location, adjuvant chemotherapy status, microsatellite instability (MSI), CpG island methylator phenotype (CIMP), *KRAS* mutation and *BRAF* mutation, to determine whether these characteristics act as confounding factors.

## Materials and Methods

### Ethics statement

The study was approved by the Institutional Ethics Committee of Seoul National University Hospital, which waived the requirement to obtain informed consent (approval No. H-1312-050-542).

### Patient specimens

We retrospectively analyzed the clinicopathologic data of the 767 CRC patients who underwent tumor resection at Seoul National University Hospital from January 2004 to December 2006. We designed a training set, which consisted of 218 CRC cases, to identify which intratumoral subsite is more reliable to evaluate the association between T cell subset densities and patient outcome. Then, a validation set, which consisted of 549 cases, was designed. We split the entire cohort into the training set and the validation set randomly. The validation set excluded all patients in the training set. Among the 767 patients, 507 were treated with adjuvant chemotherapy. Patient information such as age, gender, pathologic tumor-node-metastasis (pTNM) stage, and tumor location was obtained from electronic medical records. According to the pTNM classification (of 6^th^ version), the training set included 21 patients in stage I, 74 patients in stage II, 79 patients in stage III, and 44 patients in stage IV. The validation set included 93 patients in stage I, 174 patients in stage II, 192 patients in stage III, and 90 patients in stage IV.

### Analysis of *KRAS* and *BRAF* mutations

Manually microdissected tissues were collected into microtubes and were incubated in a mixture of lysis buffer and proteinase K at 55°C for 2 days. Direct sequencing of *KRAS* codons 12 and 13, and allele-specific polymerase chain reaction (PCR) analysis of *BRAF* codon 600 were performed as described previously [27].

### Analysis of MSI and CIMP

MSI status was determined by utilizing National Cancer Institute 5-marker scoring panel (BAT25, BAT26, D2S123, D5S345, and D17250). MSI-high was defined as the presence of instability in ≥ 40% of markers, MSI-low was defined as the presence of only one instable marker, and microsatellite stable (MSS) was defined as no instable markers.

Bisulfate DNA modification and MethyLight, a real-time PCR-based methylation assay, were performed as previously described [28]. CIMP status was determined by quantifying DNA methylation at eight markers—CACNA1G, CDKN2A (p16), CRABP1, IGF2, MLH1, NEUROG1, RUNX3, and SOCS1. CIMP-positive was defined as the presence of methylation in more than four markers, and CIMP-negative was considered when four or fewer markers were methylated.

### Tissue microarray (TMA) analysis

**Fig 1:**
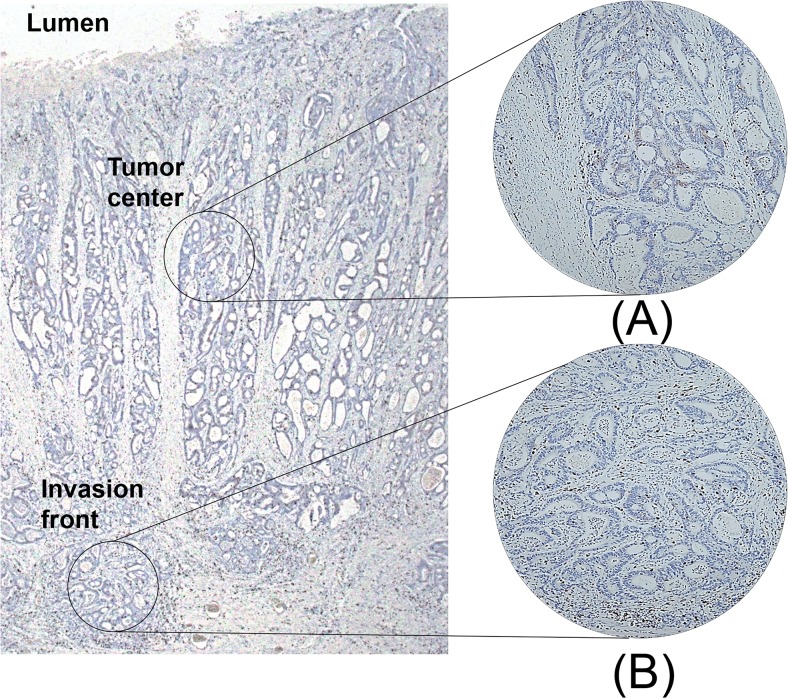
Depiction of intratumoral subsites where tissue cores were obtained. Tissue microarray (TMA) core was constructed from tumor center (A) and invasion front (B), and immunohistochemistry for CD8 was performed.

Through histologic examination, we marked portions that best represent the tumor. For the training set, one area that represents the tumor center and two areas that represent the invasion front (Fig 1) were extracted from each patient sample. For the validation set, one area that represents the invasion front was extracted from each patient sample. The cores extracted for the training set and the validation set were selected by two pathologists (YK and JMB). Core tissues (2 mm in diameter) were extracted from each paraffin-embedded CRC sample (donor block) and rearranged in a new recipient tissue microarray block using a trephine apparatus as described previously [29].

### Immunohistochemistry and image analysis

Formalin-fixed and paraffin-embedded sections of TMA were deparaffinized in xylene and were rehydrated with gradually decreasing concentrations of alcohol. Sections were immunostained after antigen retrieval using the Bond-max automated immunostainer (Leica Microsystems, Newcastle, UK). Primary antibodies used were the anti-CD8 polyclonal antibody (1:100, Neomarkers, Fremont, CA, USA), the anti-CD45RO monoclonal antibody (1:50, Neomarkers) and the anti-FOXP3 monoclonal antibody (1:50, Abcam, Cambridge, MA, USA). Antibody binding was detected by using the Bond Polymer Refine Detection kit (Leica Microsystems).

**Fig 2:**
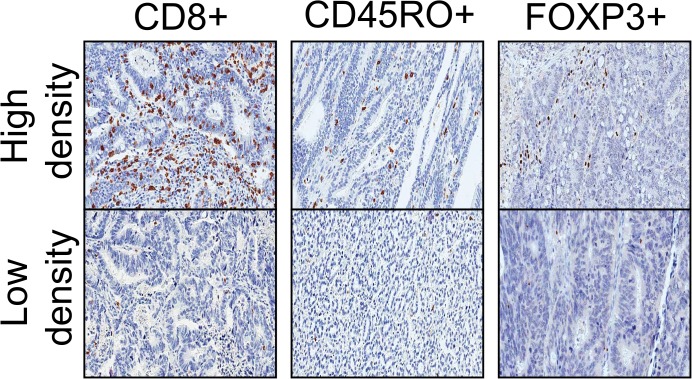
Photomicrographs of CD8, CD45RO, and FOXP3 immunohistochemistry in CRC. The panels on the top show T cell subsets with high cell densities, and the panels on the bottom show T cell subsets with low cell densities.

After immunohistochemical staining for each of the T cell markers, TMA slides were scanned by the Aperio image analysis system (Leica Biosystems, New Castle, UK) (Fig 2). The software counted the number of immunopositive nuclei in each tissue core. The average density (cells/mm^2^) of each lymphocyte subset was calculated in a whole TMA core. T cell subset densities were divided into two groups (high versus low) according to a median-split.

### Statistical analysis

All statistical analyses were performed using SPSS for Windows (version 21.0) (International Business Machines Corp., Armonk, NY, USA). The Mann-Whitney test was used to determine the relationship between T cell densities and clinicopathological parameters. We used Spearman’s correlation analysis to determine the relationship between T cell densities. The clinical database was last updated in August 2013. Progression-free survival (PFS) was calculated from the date of resection of CRC to the first date of documented recurrence or the date of death from any cause. Overall survival (OS) was measured from the date of operation to the date of death or the last clinical follow-up time before August 2013. PFS and OS were calculated according to the Kaplan-Meier method. The log-rank test was used to compare the survival distribution. Univariate and multivariate analysis were performed on the Cox regression model. Only variables that were significantly different in univariate analysis were entered into the next multivariate analysis. *p* values < 0.05 were considered statistically significant.

To avoid overfitting, twenty event per variable (EPV) was performed to limit the number of covariates in the multivariate analysis. In the training set, 76 deaths were observed, and three covariates could be used. Since up to two lymphocyte subset densities were significantly correlated with prognosis in the univariate analysis, only pTNM stage was included as the last covariate, although presence of lymphatic invasion and that of venous invasion were also significantly associated with worse prognosis (*p* < 0.001) in the training set. In the validation set, the number of death was 127, and six covariates could be used. Up to three lymphocyte subset densities were significantly correlated with prognosis in the univariate analysis, and three other covariates included pTNM stage, lymphatic invasion, and venous invasion.

## Results

### T cell subset density in CRC

**Fig 3:**
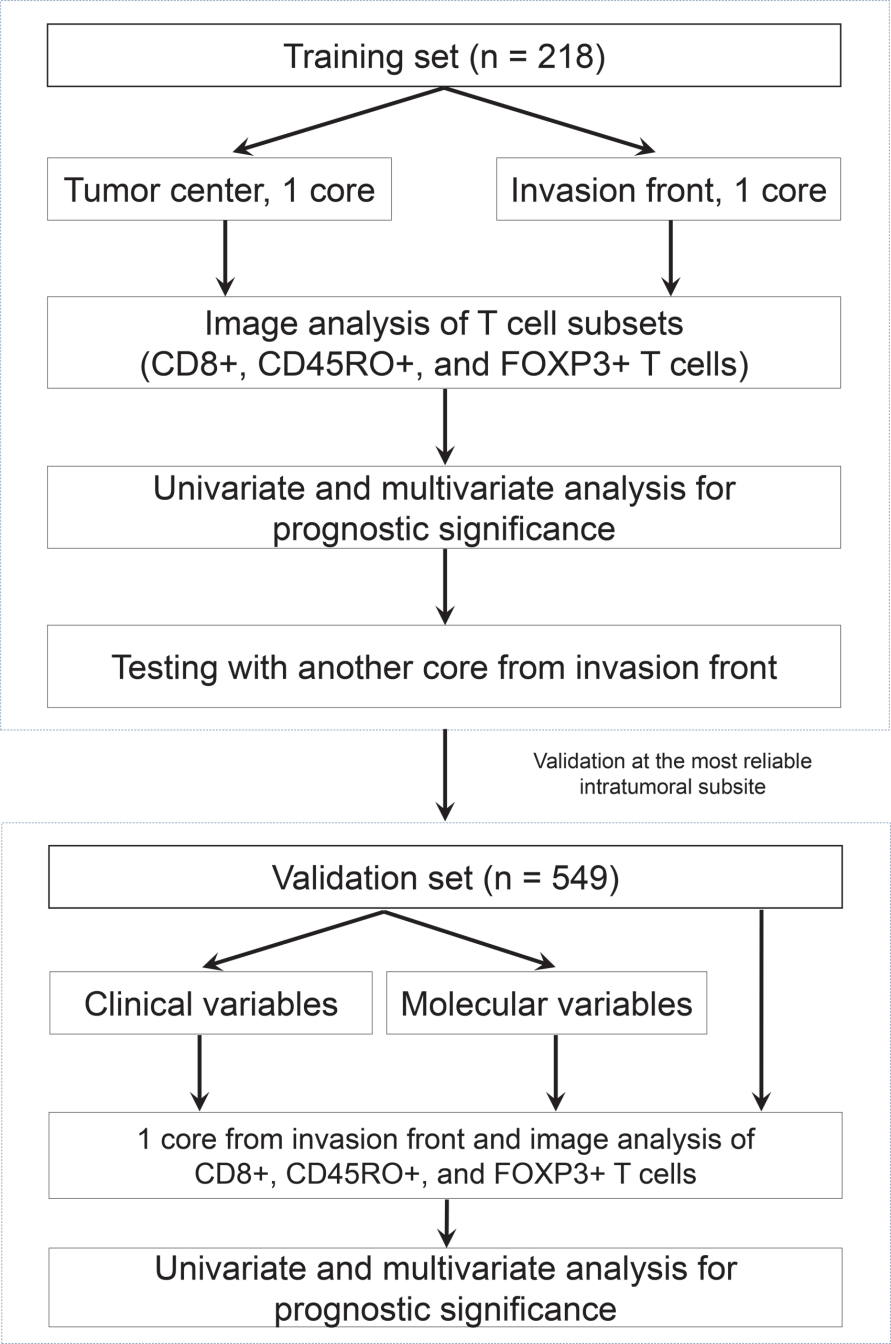
Flow chart of the study.

Using automated image analysis, we calculated CD8, CD45RO, and FOXP3 densities (cells/mm^2^) in TMA cores from the tumor center and the invasion front (see flow chart in Fig 3). The densities of CD8, CD45RO, and FOXP3 in the cores of the tumor center were 119.9, 201.8, and 50.8, respectively, and those in the invasion front were 138.3, 177.7, and 58.6, respectively. The tumor-related stroma in the invasion front (48.84 ± 21.05%) was significantly more abundant than those of the tumor center (39.32 ± 18.96%) according to Mann-Whitney test (*p* < 0.001).

### Correlation between T cell subset density and clinicopathologic characteristics in the training set

Table 1 shows the correlation between lymphocyte density and clinicopathologic variables in the tumor center and the invasion front. The median age of 218 CRC patients was 62.5 years (ranging from 28 to 85) and 61.0% of patients were male; 56.4% of tumors were pTNM stages I and II compared with 43.6% in stages III and IV. In the tumor center, none of the T cell subset densities showed significant correlation with the clinicopathological variables. In the invasion front, the CD8 density showed negative correlations with pTNM stages (*p* = 0.037) and distant metastasis (*p* = 0.002). The CD45RO density showed similar relationships (*p* = 0.012 and *p <* 0.001, respectively). In addition, the FOXP3 density was lower in colon cancer patients than in rectal cancer patients (*p* = 0.033)

**Table 1:** Relationship between T cell subset density and clinicopathological features in the training set of CRC.

		Tumor center						Invasion front					
	N (%)	CD8	*p* value	CD45RO	*p* value	FOXP3	*p* value	CD8	*p* value	CD45RO	*p* value	FOXP3	*p* value
**Age**													
<65	119 (54.6)	120.0	0.886	204.0	0.910	54.0	0.267	138.1	0.798	177.5	0.385	57.4	0.927
≥65	99 (45.4)	119.8		199.1		47.1		140.9		177.9		60.0	
**Gender**													
Male	133 (61.0)	126.3	0.371	209.3	0.875	56.4	0.071	135.9	0.627	178.2	0.740	62.5	0.087
Female	85 (39.0)	109.3		189.7		42.2		142.1		177.0		52.5	
**pTNM stage**													
I or II	123 (56.4)	128.8	0.758	214.9	0.199	47.1	0.232	154.3	0.037	212.3	0.012	57.2	0.630
III or IV	95 (43.6)	112.8		191.6		53.8		125.6		150.5		59.6	
**Lymphatic invasion**													
Yes	107 (49.1)	113.2	0.337	183.1	0.116	50.7	0.877	117.4	0.125	148.4	0.074	54.3	0.314
No	111 (50.9)	126.1		219.5		51.0		158.1		205.4		62.7	
**Distant metastasis**													
Yes	44 (20.2)	101.5	0.133	199.0	0.208	51.9	0.965	88.1	0.002	104.4	**< 0.001**	55.7	0.938
No	174 (79.8)	124.5		202.6		50.6		150.1		195.6		59.3	
**MSI status**													
Positive	8 (4.0)	130.5	0.084	154.8	0.913	46.7	0.704	241.5	0.617	226.9	0.438	56.9	0.898
Negative	190 (96.0)	119.0		205.6		51.4		137.1		178.4		5.9	
**CIMP status**													
Positive	13 (6.0)	111.7	0.843	263.4	0.227	68.2	0.130	105.2	0.249	164.5	0.540	56.7	0.927
Negative	205 (94.0)	120.5		198.1		49.7		140.5		178.5		58.7	
**Tumor location**													
Colon	158 (72.5)	122.1	0.588	195.7	0.155	49.2	0.110	144.3	0.794	179.0	0.146	56.2	**0.033**
Rectum	60 (27.5)	114.1		218.3		55.2		122.2		174.2		65.0	

T cell subset densities represent median values for labelled cells per mm^2^; MSI: microsatellite instability; CIMP: CpG island methylator phenotype

### Correlation between T cell subset densities in the training set

Table 2 shows the interrelations between the densities of T cell subsets in the tumor center and the invasion front. Spearman's correlation coefficients showed significant positive correlations between each other except for the correlation between the FOXP3 density in the tumor center and that in the invasion front (*p* = 0.165).

**Table 2:** Correlation between T cell subset density in the training set of CRC.

	Tumor center					Invasion front					
	CD8	CD45RO		FOPX3		CD8		CD45RO		FOPX3	
**Tumor center**												
**CD8**		*r* = 0.583		*r* = 0.189		*r* = 0.382		*r* = 0.262		*r* = 0.191	
		*p <* 0.001		*p* = 0.007		*p <* 0.001		*p <* 0.001		*p* = 0.007	
**CD45RO**				*r* = 0.345		*r* = 0.278		*r* = 0.420		*r* = 0.162	
				*p <* 0.001		*p <* 0.001		*p <* 0.001		*p <* 0.001	
**FOXP3**						*r* = 0.166		*r* = 0.420		*r* = 0.096	
						*p* = 0.016		*p* = 0.009		*p* = 0.165	
**Invasion front**												
**CD8**	*r* = 0.382	*r* = 0.278		*r* = 0.166							
	*p <* 0.001	*p <* 0.001		*p* = 0.016							
**CD45RO**	*r* = 0.262	*r* = 0.420		*r* = 0.420		*r* = 0.779					
	*p <* 0.001	*p <* 0.001		*p* = 0.009		*p <* 0.001					
**FOPX3**	*r* = 0.191	*r* = 0.162		*r* = 0.096		*r* = 0.506		*r* = 0.425			
	*p* = 0.007	*p <* 0.001		*p* = 0.165		*p <* 0.001		*p <* 0.001			

*r*, Spearman's correlation coefficient

### Correlation between T cell subset density and patient prognosis in the training set

**Fig 4:**
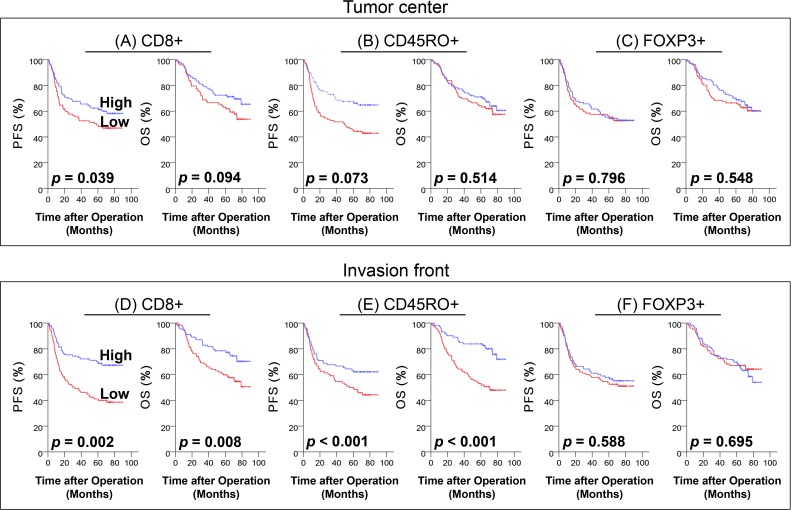
Progression free survival (PFS) and overall survival (OS) in the training set of CRC. Kaplan-Meier curves showing the prognostic significance of CD8 (A), CD45RO (B), and FOXP3 (C) densities in the tumor center and that of CD8 (D), CD45RO (E), and FOXP3 (F) densities in invasion front.

We examined the density of each T cell subset and patient survival. In Kaplan-Meier analysis with the log-rank test (Fig 4), patients with a high density of CD8 in the tumor center had significantly better PFS (*p* = 0.039; Fig 4A). In addition, high densities of CD8 (Fig 4D) and CD45RO (Fig 4E) in the invasion front were associated with favorable PFS (*p* = 0.002 and *p <* 0.001, respectively) and OS (*p* = 0.008 and *p <* 0.001, respectively).

In multivariate Cox regression analysis (Table 3), a high density of CD8 in the tumor center, and high densities of CD8 and CD45RO in the invasion front were associated with better PFS (*p* = 0.035, *p* = 0.011 and *p* = 0.001, respectively). In addition, a high density of CD45RO in the invasion front was positively correlated with a longer OS than a low density of CD45RO (*p <* 0.001).

**Table 3:** T cell subset density and CRC patient mortality in the training set.

	**Progression free survival**					
**T cell subsets**	**Univariate HR**	**95% CI**	***p* value**	**Multivariate HR**	**95% CI**	***p* value**
**Tumor center**						
CD8	0.641	0.419–0.981	**0.041**	0.632	0.413–0.967	**0.035**
CD45RO	0.691	0.461–1.038	0.075			
FOXP3	0.948	0.636–1.416	0.796			
**Invasion front**						
CD8	0.516	0.338–0.790	**0.002**	0.652	0.424–1.004	**0.011**
CD45RO	0.426	0.278–0.652	**< 0.001**	0.603	0.388–0.938	**0.001**
FOXP3	0.894	0.595–1.343	0.589			
	**Overall survival**					
**T cell subsets**	**Univariate HR**	**95% CI**	***p* value**	**Multivariate HR**	**95% CI**	***p* value**
**Tumor center**						
CD8	0.668	0.415–1.075	0.096			
CD45RO	0.860	0.546–1.354	0.515			
FOXP3	0.870	0.553–1.370	0.548			
**Invasion front**						
CD8	0.527	0.325–0.855	**0.009**	0.572	0.352–0.928	0.055
CD45RO	0.356	0.216–0.586	**< 0.001**	0.462	0.279–0.767	**< 0.001**
FOXP3	1.096	0.692–1.736	0.695			

pTNM stage was adopted as the covariate in each multivariate analysis.

To eliminate bias caused by possible tumor heterogeneity, we constructed duplicate TMA cores from the invasion front of the same CRC specimens (n = 218). CD8 and CD45RO densities were independent prognostic factors regarding PFS (*p* = 0.023 and *p* = 0.003, respectively), whereas CD45RO density was an independent prognostic factor regarding OS (*p* = 0.008) (data not shown).

### Correlation between T cell subset density and clinicopathologic characteristics in the validation set

In the training set, T cell subset densities in the invasion front showed stronger prognostic values than those in the tumor center. To gain stronger statistical power, the validation set was constructed with TMA cores from the invasive front of a larger CRC patient sample (n = 549) (see flow chart in Fig 3). Table 4 summarizes the correlation between the densities of the three T cell subsets and the clinicopathologic features of the 549 CRC patients included in the validation set. In this population, a high density of CD8 was associated with early pTNM stages (*p <* 0.001), absence of distant metastasis (*p <* 0.001), MSI-positivity (*p <* 0.001) and CIMP-positivity (*p* = 0.019). A high density of CD45RO was associated with early pTNM stages (*p <* 0.001) and absence of distant metastasis (*p <* 0.001). A high density of FOXP3 was associated with young age (*p* = 0.022), early pTNM stages (*p <* 0.001), absence of lymphatic invasion (*p* = 0.001) and absence of distant metastasis (*p <* 0.001).

**Table 4:** Relationship between T cell subset density and clinicopatholgical features in the validation set of CRC.

	N (%)	CD8	*p* value	CD45RO	*p* value	FOXP3	*p* value
**Age**							
<65	321 (58.5)	137.4	0.315	119.9	0.140	90.2	**0.022**
≥65	228 (41.5)	130.5		105.5		78.9	
**Gender**							
Male	381 (60.3)	130.7	0.063	123.5	0.395	80.8	0.074
Female	218 (39.7)	131.2		128.4		67.1	
**pTNM stage**							
I or II	267 (48.6)	159.4	**< 0.001**	135.7	**< 0.001**	104.4	**< 0.001**
III or IV	282 (51.4)	110.1		92.8		67.2	
**Lymphatic invasion**							
Yes	239 (43.5)	124.5	0.065	103.66	0.177	69.9	**0.001**
No	310 (56.5)	142.1		121.6		97.7	
**Distant metastasis**							
Yes	91 (16.6)	96.1	**< 0.001**	75.5	**< 0.001**	59.6	**< 0.001**
No	458 (83.4)	142.0		121.7		90.5	
**MSI status**							
Positive	57 (10.6)	210.4	**< 0.001**	124.2	0.354	96.8	0.085
Negative	479 (89.4)	124.9		112.3		84.0	
**CIMP status**							
Positive	35 (6.4)	197.1	**0.019**	109.5	0.932	77.09	0.262
Negative	514 (93.6)	130.4		114.24		86.1	
***KRAS*** **mutation**							
Positive	140 (27.0)	150.4	0.392	118.0	0.843	92.5	0.527
Negative	379 (73.0)	130.2		116.4		85.6	
***BRAF*** **mutation**							
Positive	25 (4.6)	154.8	0.976	143.4	0.760	84.8	0.764
Negative	519 (95.4)	133.5		122.9		85.9	
**Tumor location**							
Colon	381 (69.4)	135.4	0.720	114.8	0.292	81.4	0.116
Rectum	168 (30.6)	132.5		111.8		95.3	
**Adjuvant chemotherapy**							
Yes	351 (63.9)	125.5	0.079	105.3	0.179	77.6	0.085
No	198 (36.1)	149.9		129.0		99.3	

T cell subset densities represent median values for labelled cells per mm^2^; MSI: microsatellite instability; CIMP: CpG island methylator phenotype

### Correlation between T cell subset density and patient outcome in the validation set

**Fig 5:**
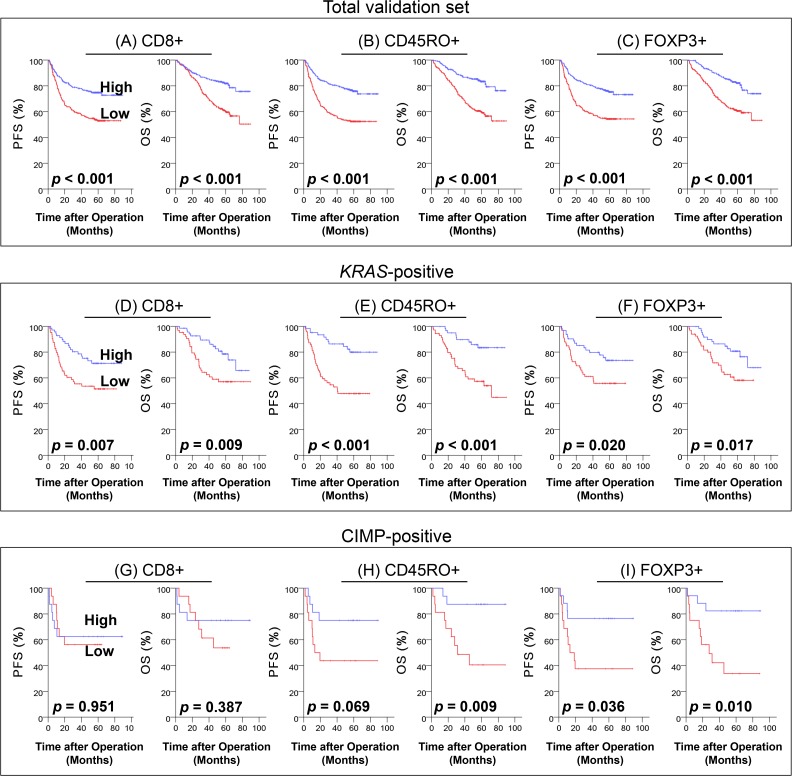
Progression free survival (PFS) and overall survival (OS) in the validation set of CRC. Kaplan-Meier curves showing the prognostic significance of CD8 (A, D and G), CD45RO (B, E and H), and FOXP3 (C, F and I) densities in invasion front from total CRC (A to C) (n = 549), *KRAS*-mutated (D to F) (n = 141), and CIMP-positive (G to I) (n = 36) patients

Kaplan-Meier analysis with the log-rank test showed significant correlations between all three T cell subset densities and PFS as well as OS for the total CRC patients (*p <* 0.001) (Fig 5A–5C). When patient samples were divided into two groups according to tumor location (colon versus rectum) or adjuvant chemotherapy status, all four groups showed positive correlations between all three T cell subset densities and PFS as well as OS (S1 Fig). Furthermore, patient samples were divided according to molecular variables (MSI, CIMP, *KRAS* mutation, and *BRAF* mutation). None of the three T cell subset densities were significantly associated with PFS and OS in MSI-high and *BRAF*-mutated samples (S2 Fig). By contrast, analysis of *KRAS*-mutated samples (Fig 5D–5F) showed that CD8+, CD45RO+, and FOXP3+ T cell subset densities were positively correlated with PFS (*p* = 0.007, *p <* 0.001 and *p* = 0.020, respectively) and OS (*p* = 0.009, *p <* 0.001 and *p* = 0.017, respectively). In CIMP-positive specimens (Fig 5G–5I), the FOXP3 density was positively correlated with PFS (*p* = 0.036), and CD45RO and FOXP3 densities were positively correlated with OS (*p* = 0.009 and *p* = 0.010, respectively).

In multivariate analysis, the CD45RO density was positively correlated with PFS and OS in CRC patients (*p* = 0.011 and *p* = 0.008, respectively; Table 5). This was also true when the group was divided into colon cancer (*p* = 0.040 and *p* = 0.049, respectively) and rectal cancer patients (*p* = 0.035 and *p* = 0.002, respectively; S1 Table). In patients who did not receive postoperative chemotherapy, CD8, and FOXP3 densities showed positive correlations with OS (*p* = 0.011; S2 Table). The CD45RO density was an independent prognostic factor regardless of chemotherapy status (S2 Table). Although CD45RO density was significant for PFS (*p* = 0.003) and OS (*p* = 0.004) in *KRAS*-mutated specimens (S3 Table), other T cell subset densities were not independent prognostic factors in *KRAS*-mutated (S3 Table) and CIMP-positive specimens (S4 Table).

**Table 5:** T cell subset density and patient outcome in the validation set of CRC.

	**Progression free survival**					
**T cell subsets**	**Univariate HR**	**95% CI**	***p* value**	**Multivariate HR**	**95% CI**	***p* value**
CD8	0.477	0.351–0.648	**< 0.001**	0.851	0.568–1.277	0.436
CD45RO	0.420	0.307–0.576	**< 0.001**	0.549	0.363–0.830	**0.011**
FOXP3	0.453	0.329–0.623	**< 0.001**	0.462	0.564–1.297	0.462
	**Overall survival**					
**T cell subsets**	**Univariate HR**	**95% CI**	***p* value**	**Multivariate HR**	**95% CI**	***p* value**
CD8	0.426	0.300–0.606	**< 0.001**	0.952	0.602–1.506	0.834
CD45RO	0.361	0.251–0.520	**< 0.001**	0.470	0.293–0.753	**0.007**
FOXP3	0.403	0.281–0.577	**< 0.001**	0.704	0.448–1.109	0.130

pTNM stage, lymphatic invasion, venous invasion, and all three T cell subset densities were adopted as covariates in each multivariate analysis

## Discussion

The present study investigated the prognostic significance of T cell subset (CD8, CD45RO, and FOXP3) densities using TMA cores and compared the difference between the tumor center and the invasion front of CRC. Furthermore, we used 2 mm diameter cores, which is at least 4 times larger than TMA cores (0.6 mm to 1 mm diameter) used in previous studies [11,19,21,26] to exclude the possible effects of tumor heterogeneity. Computerized image analysis software was used to minimize observer bias. In the training set composed of 218 CRC specimens, we found that T cell subset densities in the invasion front were stronger prognostic factors than those in the tumor center. In the validation set with 549 CRC specimens, all three T cell subset densities in the invasion front were associated with PFS and OS in univariate analysis. In multivariate analysis, only the CD45RO density in the invasion front was a prognostic factor in CRC, independent of pTNM stage, lymphatic invasion, venous invasion, and other T cell subset (CD8 and FOXP3) densities.

In previous reports, CD8+ T cells were correlated with prognosis in a univariate analysis of CRC patients [22,25], but not in a multivariate analysis. CD8/FOXP3, on the other hand, was proven to be an independent prognostic factor in a single study with a small sample size (n = 94) [30]. We found that, in multivariate analysis, a high CD8 density was correlated with good OS in chemotherapy-free CRC patients.

In CRC patients, a strong infiltration of CD45RO+ T cells was associated with good OS or disease free survival (DFS) in three published studies [19,22,31]. Among them, two studies were limited to early-stage CRC patients [19,31]. Meanwhile, Nosho et *al*. [22] did not stratify adjuvant chemotherapy status of the CRC patients. In the present study, we used CRC specimens from stage I to IV and found that the CD45RO density was an independent prognostic factor in patients with CRC, regardless of tumor location or adjuvant chemotherapy status. CD45RO is expressed on both memory and effector T cells. Since additional markers such as CCR7 are necessary to differentiate those populations, immunohistochemical staining using anti-CCR7 antibody was performed on the TMA for the training set. We found that CCR7 expression showed no significant correlation with patient prognosis in the univariate analysis (data not shown).

There have been a number of inconsistent reports regarding the relationship between the density of FOXP3+ T cells and patient survival in CRC. Several studies reported tumor infiltrating FOPX3+ T cell density as an unfavorable prognostic factor in CRC or advanced colon cancer [21,23]. Others claimed that FOXP3+ T cells was a favorable prognostic factor in CRC [32] and colon cancer [31,33] or was not an independent prognostic factor of CRC [22]. This discrepancy could be explained by hypothesizing an ambivalent relationship between FOXP3+ T cells and CRC. FOPX3+ T cells are known to suppress Th1-dependent antitumor immune response in several solid tumors; however, FOXP3+ T cells in septic environments induce an antitumor immune response by attenuating bacteria-stimulated Th17 cells [15]. If both of these reactions occur in CRC, inconsistency in the prognostic impact of FOXP3 might be caused by confounding factors affecting the role of FOXP3+ T cells in an antitumor immune response. Possible confounding factors include CD45RO+ T cells and adjuvant chemotherapy status. If this hypothesis is valid, the FOXP3 density might not be an appropriate prognostic factor in CRC.

In a recent meta-analysis study, Mei et *al*. [34] showed that CD8+ and FOXP3+ T cells were not significant prognostic markers in the tumor center of CRC. However, they could not evaluate CD45RO+ T cells because of the limited number of published studies. In addition, they suggested that clinical and molecular factors affecting the tumor microenvironment of CRC should be further evaluated. To this end, we adjusted clinical variables (tumor location and adjuvant chemotherapy) as well as key molecular variables associated with evolution of CRC (MSI, CIMP, *KRAS* mutation, and *BRAF* mutation) [35]. Unlike Nosho et *al*. [22], the present study did not show a prognostic significance of CD45RO in MSI-high patients but showed a prognostic significance in *KRAS*-mutated patients. This discrepancy might reflect different proportions of molecular variables in CRC, which commonly appears between Eastern and Western populations [28,36–39].

In conclusion, we found that among the three T cell subsets evaluated, only the CD45RO+ T cell density was significantly associated with longer survival in CRC patients, independent of tumor stage, lymphovascular invasion, and CD8+ and FOXP3+ T cell densities. However, CD8 and FOXP3 densities were also independent prognostic factors in certain clinical settings. Our results suggest that measuring T cell subset densities by automated image analysis of TMA cores in the invasion front, rather than those in the tumor center, is a more reliable strategy for predicting prognosis in CRC patients. Therefore, increasing T cell subsets within tumors through therapeutic intervention may benefit CRC patients with prolonged survival.

## Supporting Information

S1 Fig Progression free survival (PFS) and overall survival (OS) in the validation set according to tumor location and chemotherapy status. Kaplan-Meier curves showing the prognostic significance of CD8 (A, D, G and J), CD45RO (B, E,H, and K), and FOXP3 (C, F, I, and L) densities in the invasion front from colon cancer (A to C), rectal cancer (D to F), chemotherapy-treated (G to I), and chemotherapy-free (J to L) patients.
(TIF)

S2 Fig Progression free survival (PFS) and overall survival (OS) in the validation set of MSI-high and *BRAF*-mutated CRC. Kaplan-Meier curves showing the prognostic significance of CD8 (A), CD45RO (B), and FOXP3 (C) densities in MSI-high and that of CD8 (D), CD45RO (E), FOXP3 (F) densities in *BRAF*-mutated CRC specimens.
(TIF)

S1 Table T cell subset density and patient outcome in colon and rectal cancer patients. (DOCX)

S2 Table T cell subset density and patient outcome in CRC patients with or without adjuvant chemotherapy. (DOCX)

S3 Table T cell subset density and patient outcome in *KRAS*-mutated CRC specimens. (DOCX)

S4 Table T cell subset density and patient outcome in CIMP-positive CRC specimens. (DOCX)
