# Supplementary figures and images for: Image Analyzer-Based Assessment of Tumor-Infiltrating T Cell Subsets and Their Prognostic Values in Colorectal Carcinomas
Source: PLoS One. 2015 Apr 15;10(4):e0122183. doi: 10.1371/journal.pone.0122183 (PMC4398542; doi:10.1371/journal.pone.0122183)

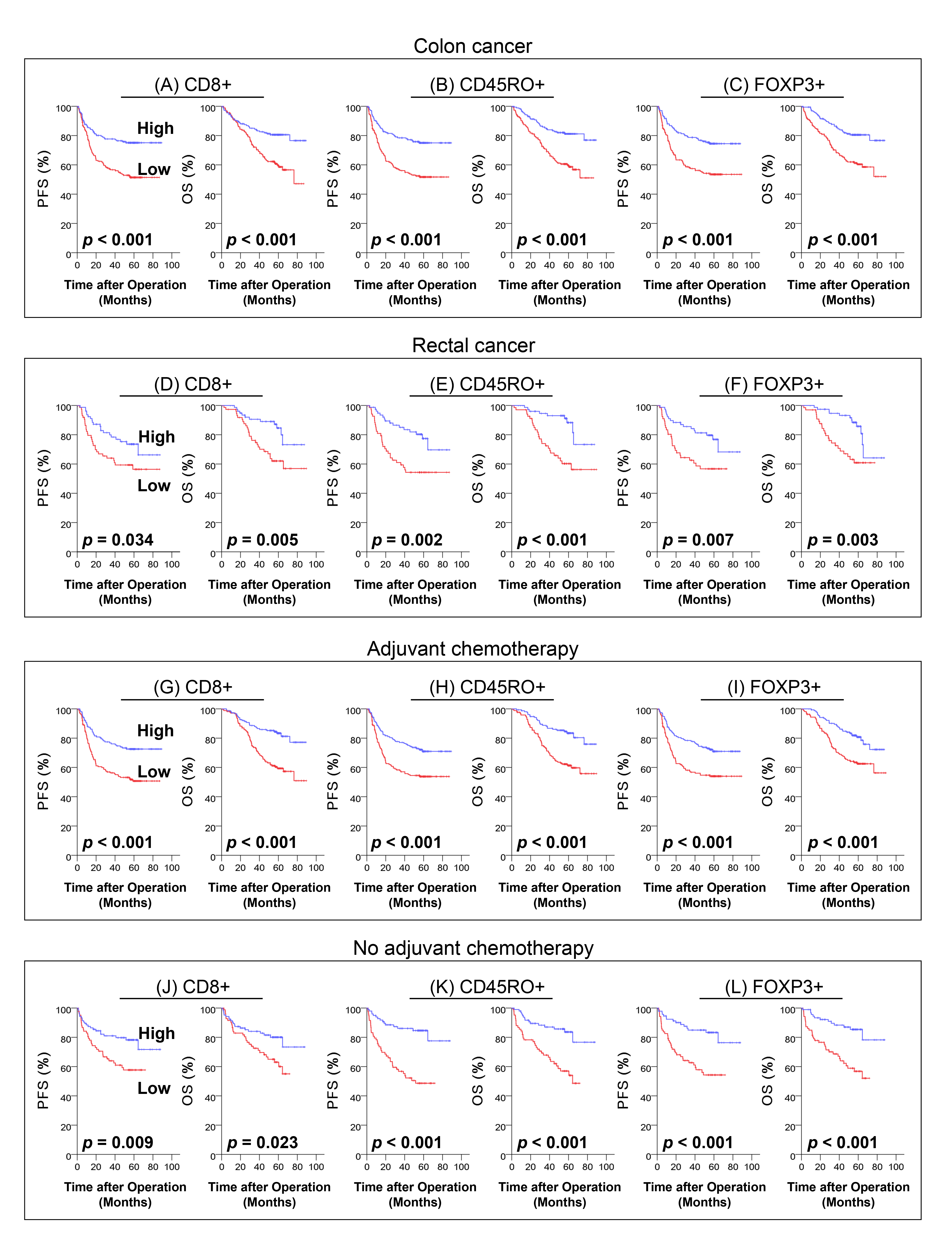

Supplement: S1 Fig — Kaplan-Meier curves showing the prognostic significance of CD8 (A, D, G and J), CD45RO (B, E,H, and K), and FOXP3 (C, F, I, and L) densities in the invasion front from colon cancer (A to C), rectal cancer (D to F), chemotherapy-treated (G to I), and chemotherapy-free (J to L) patients. (TIF) [file pone.0122183.s001.tif]

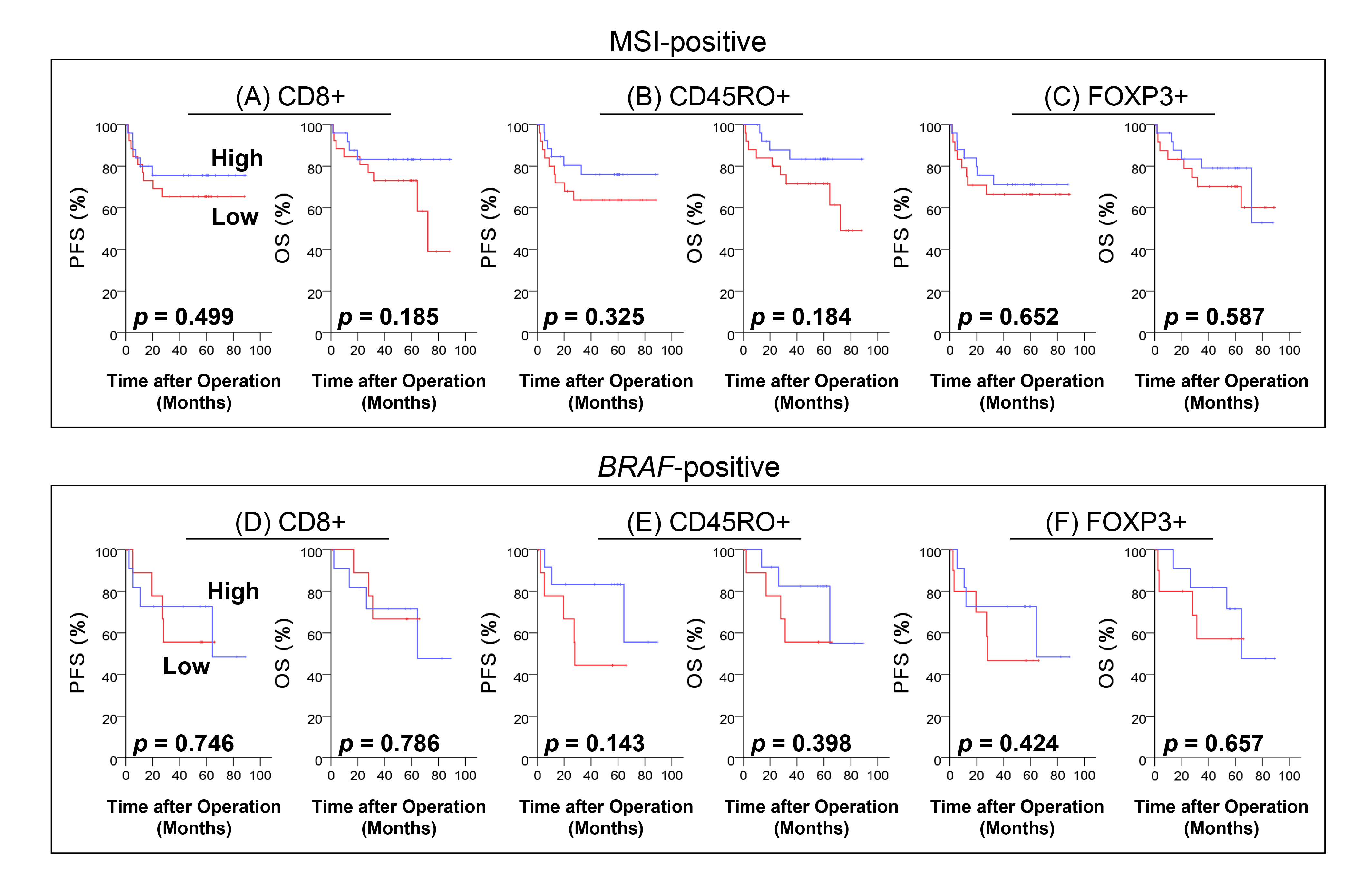

Supplement: S2 Fig — Kaplan-Meier curves showing the prognostic significance of CD8 (A), CD45RO (B), and FOXP3 (C) densities in MSI-high and that of CD8 (D), CD45RO (E), FOXP3 (F) densities in BRAF-mutated CRC specimens. (TIF) [file pone.0122183.s002.tif]
