# Supplementary material for: Image Analyzer-Based Assessment of Tumor-Infiltrating T Cell Subsets and Their Prognostic Values in Colorectal Carcinomas
Source: PLoS One. 2015 Apr 15;10(4):e0122183. doi: 10.1371/journal.pone.0122183 (PMC4398542; doi:10.1371/journal.pone.0122183)
Supplement: S1 Table — (DOCX) [file pone.0122183.s003.docx]

**Table S1. T cell subset density and patient outcome in colon and rectal cancer patients.**

| **Colon cancer** |  | | |  |  |  |
| --- | --- | --- | --- | --- | --- | --- |
|  | **Progression free survival** | | |  |  |  |
| **T cell subsets** | **Univariate HR** | **95 % CI** | ***p* value** | **Multivariate HR** | **95 % CI** | ***p* value** |
| CD8 | 0.432 | 0.287-0.651 | **< 0.001** | 0.701 | 0.427-1.15 | 0.159 |
| CD45RO | 0.393 | 0.26-0.596 | **< 0.001** | 0.605 | 0.375-0.978 | **0.040** |
| FOXP3 | 0.425 | 0.279-0.647 | **< 0.001** | 0.880 | 0.541-1.431 | 0.607 |
|  | **Overall survival** |  |  |  |  |  |
| **T cell subsets** | **Univariate HR** | **95 % CI** | ***p* value** | **Multivariate HR** | **95 % CI** | ***p* value** |
| CD8 | 0.443 | 0.294-0.667 | **< 0.001** | 0.815 | 0.473-1.406 | 0.462 |
| CD45RO | 0.400 | 0.264-0.607 | **< 0.001** | 0.585 | 0.344-0.996 | **0.049** |
| FOXP3 | 0.990 | 0.986-0.995 | **< 0.001** | 0.674 | 0.402-1.133 | 0.137 |
| **Rectal Cancer** |  | | |  |  |  |
|  | **Progression free survival** | | |  |  |  |
| **T cell subsets** | **Univariate HR** | **95 % CI** | ***p* value** | **Multivariate HR** | **95 % CI** | ***p* value** |
| CD8 | 0.547 | 0.311-0.964 | **0.037** | 1.574 | 0.73-3.393 | 0.247 |
| CD45RO | 0.390 | 0.212-0.715 | **0.002** | 0.368 | 0.145-0.93 | **0.035** |
| FOXP3 | 0.443 | 0.241-0.814 | **0.009** | 0.951 | 0.366-2.474 | 0.918 |
|  | **Overall survival** |  |  |  |  |  |
| **T cell subsets** | **Univariate HR** | **95 % CI** | ***p* value** | **Multivariate HR** | **95 % CI** | ***p* value** |
| CD8 | 0.389 | 0.197-0.769 | **0.007** | 1.792 | 0.72-4.461 | 0.210 |
| CD45RO | 0.267 | 0.125-0.571 | **< 0.001** | 0.172 | 0.056-0.53 | **0.002** |
| FOXP3 | 0.362 | 0.18-0.729 | **0.004** | 1.073 | 0.372-3.095 | 0.896 |

pTNM stage, lymphatic invasion, venous invasion, and all three T cell subset densities were adopted as covariates in each multivariate analysis
