# Supplementary material for: Image Analyzer-Based Assessment of Tumor-Infiltrating T Cell Subsets and Their Prognostic Values in Colorectal Carcinomas
Source: PLoS One. 2015 Apr 15;10(4):e0122183. doi: 10.1371/journal.pone.0122183 (PMC4398542; doi:10.1371/journal.pone.0122183)
Supplement: S2 Table — (DOCX) [file pone.0122183.s004.docx]

**Table S2. T cell subset density and patient outcome in CRC patients with or without adjuvant chemotherapy.**

| **CRC with chemotherapy** | | | |  |  |  |
| --- | --- | --- | --- | --- | --- | --- |
|  | **Progression free survival** | | |  |  |  |
| **T cell subsets** | **Univariate HR** | **95 % CI** | ***p* value** | **Multivariate HR** | **95 % CI** | ***p* value** |
| CD8 | 0.478 | 0.328-0.695 | **< 0.001** | 0.650 | 0.405-1.041 | 0.073 |
| CD45RO | 0.530 | 0.365-0.771 | **< 0.001** | 0.635 | 0.393-1.025 | 0.063 |
| FOXP3 | 0.528 | 0.362-0.771 | **< 0.001** | 1.038 | 0.631-1.708 | 0.883 |
|  | **Overall survival** |  |  |  |  |  |
| **T cell subsets** | **Univariate HR** | **95 % CI** | ***p* value** | **Multivariate HR** | **95 % CI** | ***p* value** |
| CD8 | 0.373 | 0.237-0.587 | **< 0.001** | 0.651 | 0.371-1.142 | 0.134 |
| CD45RO | 0.382 | 0.242-0.603 | **< 0.001** | 0.482 | 0.273-0.853 | **0.012** |
| FOXP3 | 0.472 | 0.305-0.731 | **< 0.001** | 0.932 | 0.54-1.611 | 0.802 |
| **CRC without chemotherapy** | | | |  |  |  |
|  | **Progression free survival** | | |  |  |  |
| **T cell subsets** | **Univariate HR** | **95 % CI** | ***p* value** | **Multivariate HR** | **95 % CI** | ***p* value** |
| CD8 | 0.493 | 0.287-0.846 | **0.010** | 2.108 | 0.937-4.738 | 0.071 |
| CD45RO | 0.259 | 0.143-0.469 | **< 0.001** | 0.277 | 0.121-0.637 | **0.002** |
| FOXP3 | 0.323 | 0.176-0.59 | **< 0.001** | 0.525 | 0.233-1.181 | 0.119 |
|  | **Overall survival** |  |  |  |  |  |
| **T cell subsets** | **Univariate HR** | **95 % CI** | ***p* value** | **Multivariate HR** | **95 % CI** | ***p* value** |
| CD8 | 0.525 | 0.298-0.924 | **0.025** | 3.061 | 1.29-7.265 | **0.011** |
| CD45RO | 0.318 | 0.174-0.583 | **< 0.001** | 0.318 | 0.133-0.759 | **0.010** |
| FOXP3 | 0.299 | 0.158-0.568 | **< 0.001** | 0.339 | 0.148-0.778 | **0.011** |

pTNM stage, lymphatic invasion, venous invasion, and all three T cell subset densities were adopted as covariates in each multivariate analysis
