# Supplementary material for: Image Analyzer-Based Assessment of Tumor-Infiltrating T Cell Subsets and Their Prognostic Values in Colorectal Carcinomas
Source: PLoS One. 2015 Apr 15;10(4):e0122183. doi: 10.1371/journal.pone.0122183 (PMC4398542; doi:10.1371/journal.pone.0122183)
Supplement: S3 Table — (DOCX) [file pone.0122183.s005.docx]

**Table S3. T cell subset density and patient outcome in *KRAS*-mutated CRC specimens.**

| ***KRAS*-mutated** |  | | |  |  |  |
| --- | --- | --- | --- | --- | --- | --- |
|  | **Progression free survival** | | |  |  |  |
| **T cell subsets** | **Univariate HR** | **95 % CI** | ***p* value** | **Multivariate HR** | **95 % CI** | ***p* value** |
| CD8 | 0.455 | 0.254-0.818 | **0.008** | 1.102 | 0.484-2.509 | 0.818 |
| CD45RO | 0.269 | 0.136-0.531 | **< 0.001** | 0.294 | 0.132-0.654 | **0.003** |
| FOXP3 | 0.482 | 0.257-0.904 | **0.023** | 0.853 | 0.374-1.948 | 0.706 |
|  | **Overall survival** |  |  |  |  |  |
| **T cell subsets** | **Univariate HR** | **95 % CI** | ***p* value** | **Multivariate HR** | **95 % CI** | ***p* value** |
| CD8 | 0.440 | 0.232-0.833 | **0.012** | 1.157 | 0.466-2.874 | 0.754 |
| CD45RO | 0.276 | 0.131-0.583 | **0.001** | 0.281 | 0.119-0.663 | **0.004** |
| FOXP3 | 0.450 | 0.229-0.882 | **0.020** | 0.767 | 0.304-1.931 | 0.573 |

pTNM stage, lymphatic invasion, venous invasion and all three T cell subset densities were adopted as covariates in each multivariate analysis
