# Supplementary material for: Image Analyzer-Based Assessment of Tumor-Infiltrating T Cell Subsets and Their Prognostic Values in Colorectal Carcinomas
Source: PLoS One. 2015 Apr 15;10(4):e0122183. doi: 10.1371/journal.pone.0122183 (PMC4398542; doi:10.1371/journal.pone.0122183)
Supplement: S4 Table — (DOCX) [file pone.0122183.s006.docx]

**Table S4. T cell subset density and patient outcome in CIMP-positive CRC specimens.**

| **CIMP-positive** |  | | |  |  |  |
| --- | --- | --- | --- | --- | --- | --- |
|  | **Progression free survival** | | |  |  |  |
| **T cell subsets** | **Univariate HR** | **95 % CI** | ***p* value** | **Multivariate HR** | **95 % CI** | ***p* value** |
| CD8 | 0.966 | 0.324-2.877 | 0.951 |  |  |  |
| CD45RO | 0.351 | 0.108-1.142 | 0.082 |  |  |  |
| FOXP3 | 0.988 | 0.975-1.002 | 0.097 |  |  |  |
|  | **Overall survival** |  |  |  |  |  |
| **T cell subsets** | **Univariate HR** | **95 % CI** | ***p* value** | **Multivariate HR** | **95 % CI** | ***p* value** |
| CD8 | 0.584 | 0.17-2.003 | 0.393 |  |  |  |
| CD45RO | 0.165 | 0.035-0.769 | **0.022** | 0.228 | 0.045-1.157 | 0.074 |
| FOXP3 | 0.210 | 0.057-0.769 | **0.018** | 0.992 | 0.977-1.007 | 0.312 |

pTNM stage was adopted as covariate in each multivariate analysis
